# Clinical Management and Burden of Prostate Cancer: A Markov Monte Carlo Model

**DOI:** 10.1371/journal.pone.0113432

**Published:** 2014-12-04

**Authors:** Chiranjeev Sanyal, Armen Aprikian, Fabio Cury, Simone Chevalier, Alice Dragomir

**Affiliations:** 1 Department of Surgery, Division of Urology, McGill University, Montreal, Quebec, Canada; 2 Research Institute of McGill University Health Center, Montreal, Quebec, Canada; 3 Department of Radiation Oncology, McGill University Health Center, Montreal, Quebec, Canada; University of Kentucky College of Medicine, United States of America

## Abstract

**Background:**

Prostate cancer (PCa) is the most common non-skin cancer among men in developed countries. Several novel treatments have been adopted by healthcare systems to manage PCa. Most of the observational studies and randomized trials on PCa have concurrently evaluated fewer treatments over short follow-up. Further, preceding decision analytic models on PCa management have not evaluated various contemporary management options. Therefore, a contemporary decision analytic model was necessary to address limitations to the literature by synthesizing the evidence on novel treatments thereby forecasting short and long-term clinical outcomes.

**Objectives:**

To develop and validate a Markov Monte Carlo model for the contemporary clinical management of PCa, and to assess the clinical burden of the disease from diagnosis to end-of-life.

**Methods:**

A Markov Monte Carlo model was developed to simulate the management of PCa in men 65 years and older from diagnosis to end-of-life. Health states modeled were: risk at diagnosis, active surveillance, active treatment, PCa recurrence, PCa recurrence free, metastatic castrate resistant prostate cancer, overall and PCa death. Treatment trajectories were based on state transition probabilities derived from the literature. Validation and sensitivity analyses assessed the accuracy and robustness of model predicted outcomes.

**Results:**

Validation indicated model predicted rates were comparable to observed rates in the published literature. The simulated distribution of clinical outcomes for the base case was consistent with sensitivity analyses. Predicted rate of clinical outcomes and mortality varied across risk groups. Life expectancy and health adjusted life expectancy predicted for the simulated cohort was 20.9 years (95%CI 20.5–21.3) and 18.2 years (95% CI 17.9–18.5), respectively.

**Conclusion:**

Study findings indicated contemporary management strategies improved survival and quality of life in patients with PCa. This model could be used to compare long-term outcomes and life expectancy conferred of PCa management paradigms.

## Introduction

Prostate Cancer (PCa) is the most common non-skin cancer and among leading cause of cancer mortality in men in developed countries. [1] In 2013, the age-standardized incidence and mortality rates in Canada were estimated at 103.9 and 17.8 per 100,000, respectively. [2] Further, most men diagnosed with PCa was aged 65 years and older. [2] Various classification systems exist to stratify patients into low, intermediate, and high risks. [3] A range of curative treatment choices are used to manage the disease by risk groups at diagnosis, from diagnosis to end-of-life. Beside active surveillance for low risk cancer, initial treatments with curative intent include radical prostatectomy and radiation therapy. Moreover, treatment options, such as hormonal manipulation, chemotherapy, and palliative radiation, are used to manage patients with advanced stages of the disease including metastatic castrate resistant prostate cancer (mCRPC). Treatment choices for the initial and advanced stages of the disease are aimed at prolonging survival and improve quality of life. However, these treatments entail uncertainty on risks and benefits that require complex clinical decision making to attain anticipated outcomes in patients [4]–[8].

Over the years, there has been growing use of decision analytic models or mathematical frameworks for evidence-informed decision making. Decision analytic models facilitate the quantitative synthesis of evidence on survival and other clinical outcomes of medical interventions over short and long term periods. The existing literature is limited on decision analytic models for the clinical management of PCa and outcomes in contemporary setting from diagnosis to end-of-life.[9]–[11] Moreover these models precede [9]–[11] the adoption of newer treatments or health technologies by healthcare systems, such as active surveillance and intensity modulated radiation therapy. [8], [12] Systemic treatments for advanced stage of the disease were also not considered by preceding models.[5], [6], [8]–[11] As a result, existing decision analytic models have not assessed the survival and other clinical outcomes attained by contemporary management options and its bearing on clinical burden of the disease.[9]–[11] To date, there is lack of randomized clinical trials that have concurrently evaluated the survival and other outcomes (e.g. recurrence or mCRPC) associated with active surveillance, radical prostatectomy, brachytherapy and intensity modulated radiation therapy. Further, concurrent assessment of all contemporary treatments by RCT’s are challenged by ethical issues, expensive/resource intensive endeavour, highly selective subjects (inclusion/exclusion criteria) unrepresentative of clinical practice, and often conducted over short follow up. [13] In view of these limitations an up-to-date decision analytic model is needed to integrate the role of contemporary management strategies on the clinical burden of the disease. The objectives of this study were to develop and validate a Markov Monte Carlo model for the contemporary clinical management of PCa, and to assess the clinical burden of the disease from diagnosis to end-of-life.

## Methods

**Figure 1:**
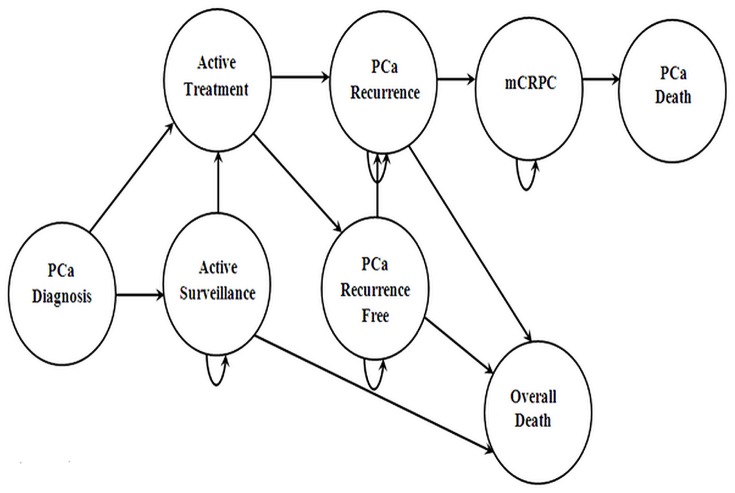
Schematic diagram of the computer simulation model. For each simulation, patients transited from left to right of the model. Incident PCa cases were distributed to active surveillance or curative intent initial treatment ascertained by level of risk at diagnosis. Straight arrows indicated potential transition pathways over successive cycles. Curved arrows indicated cases remained on that health state over successive cycles. Transition between health states was ascertained by state transition probabilities and disease evolution. Following active surveillance or initial treatment, patients were subsequently treated for PCa recurrence and metastatic castration resistant prostate cancer (i.e. mCRPC) ascertained by state transition probabilities and disease evolution over successive cycles. Patients deceased from PCa or other causes exited the model.

A Markov model with Monte Carlo microsimulation was developed to simulate the evolution of the disease, its management and associated clinical outcomes in the contemporary context. [14] Figure 1 represents the proposed model with eight distinct health states from diagnosis to end-of-life. A hypothetical annual cohort of incident cases of men 65 years and older in Canada (n = 14,160) was simulated over a 5-, 10-, 15-year and lifetime period. [2] The sample size of the low, intermediate, and high risk groups were 7080, 4248, and 2832, respectively. [2] This state-transition model with microsimulation enabled flexible modeling of the evolution of the disease and treatment choices at an individual patient level.

### Health states in the model

Hypothetical patients with PCa were simulated to receive active surveillance or active treatments as ascertained by level of risk at diagnosis. These patients based on disease evolution (or not), were transitioned to PCa recurrence free, or received treatments for recurrence, mCRPC, and finally die during the simulation from PCa or other causes. Figure 1 illustrates the eight distinct health states transit during the simulated period:

- ‘PCa diagnosis’, incident cases stratified into low, intermediate, and high risk groups.
- ‘Active surveillance’, eligible low risk patients underwent surveillance. During the simulation if the disease progressed they underwent radical prostatectomy or radiation therapy with (or without) androgen deprivation therapy. Otherwise, they were free of disease progression and died from other causes [8].
- ‘Active treatment’, eligible patients in all risk groups received curative intent active (initial) treatment (i.e. radical prostatectomy or radiation therapy with/or without androgen deprivation therapy). [8] The clinical literature indicated similar clinical outcomes attained by open or robotic surgical approaches. [8], [15], [16] Internal radiation therapy (i.e. brachytherapy) and external beam radiation therapy (i.e. intensity modulated radiation therapy) were simulated by the model.
- ‘PCa recurrence’, represents disease recurrence following failure of initial treatments that triggered initiation of subsequent treatment. [8] Patients who transited to PCa recurrence remained in this state till they progressed to mCRPC or died from other causes.
- ‘PCa recurrence free’, represents disease recurrence free following initial treatments.
- ‘mCRPC’, represents the metastatic castrate resistant state of the disease following failure of subsequent therapy. Patients were simulated to receive systemic treatments to improve survival [5], [8].
- ‘PCa death’, represents death from PCa. Patients who transited to mCRPC state remained in that state till they progressed to PCa death [7].
- ‘Overall death’, represents death from other competing causes. Patients on active surveillance, PCa recurrence, and PCa recurrence-free progressed to overall death during the simulation based on state transition probability.

### Treatment options simulated by risk groups

Evolution of the disease was simulated based on level of risk at diagnosis. Hence, following treatment options were simulated based on level of risk at diagnosis:

- Low risk – eligible patients were simulated to receive either active surveillance followed by delayed treatment (i.e. radical prostatectomy or radiation therapy) or curative intent treatment (i.e. radical prostatectomy or radiation therapy) at diagnosis. Patients were simulated to receive intensity modulated radiation therapy or brachytherapy [8].
- Intermediate risk – these patients were simulated to receive either radical prostatectomy or radiation therapy at diagnosis. Patients were simulated to receive intensity modulated radiation therapy as monotherapy or in combination with brachytherapy or androgen deprivation therapy. The median duration of ADT use was 8 months [8], [17].
- High risk – these patients were simulated to receive intensity modulated radiation therapy and androgen deprivation therapy with (or without) brachytherapy. The median duration of ADT use was 15 months [8], [17].

Following the failure of initial treatments (i.e. cancer recurrence), patients from all risk groups were simulated to received subsequent treatments. Subsequent treatment simulated following the failure of initial treatment with radical prostatectomy was radiation therapy with (or without) androgen deprivation therapy. Further, following the failure of initial radiation therapy patients were simulated to receive androgen deprivation as subsequent treatment [8]. State transition probabilities for subsequent treatments were ascertained during simulation.

### State-transition probabilities

The state-transition probabilities used to develop the model were derived from peer- reviewed literature [17]–[22]. Study findings were reported as rates over a time period (i.e. cumulative incidence). These were converted to annual rates followed by annual probabilities. Annual rates (r_1y_) were derived using the formula r_1y_ = −[ln (1–r)/t], where ‘r’ was the rate reported by studies and ‘t’ was the time period corresponding to the rate. Annual probabilities of the event (p_1y_) were derived from annual rates using the formula p_1y_ = 1-exp (−r_1y_), where ‘p_1y_’ was the annual probability and ‘r_1y_’ was the annual rate. [14] Health states transited by patients during the simulated periods were counted by tracker variables [23].

### Model overview and assumptions

Initial treatment distributions were adapted from peer-reviewed literature that reflected the clinical practice in Quebec, Canada.[17]–[22] Simulated patients were assigned to initial treatments specific to level of risk at diagnosis. In the low risk cohort, 10% were assumed to undergo active surveillance and 90% were assumed to receive initial treatments. [24], [25] Patients on active surveillance were assumed to receive a delayed treatment at an annual probability of 0.08 for first 2 years, 0.04 for 3 to 5 years, and 0.02 for 5 to 10 years. [18] The 90% of patients simulated to receive curative intent treatment were distributed as follows: 0.30 for radical prostatectomy, 0.30 for intensity modulated radiation therapy, and 0.30 for brachytherapy. [21], [26] In contrast, intermediate and high risk patients were assumed to receive a curative intent initial treatment following diagnosis. The distribution of initial treatments received by intermediate risk cohort was 0.49 for radical prostatectomy, 0.24 for intensity modulated radiation therapy, 0.19 for intensity modulated radiation therapy+androgen deprivation therapy, and 0.08 for intensity modulated radiation therapy+brachytherapy. [17], [20], [27] The distribution of initial treatments received by high risk cohort was 0.77 for intensity modulated radiation therapy+androgen deprivation therapy and 0.23 for intensity modulated radiation therapy+androgen deprivation therapy+brachytherapy [17], [20].

The disease management trajectory for low, intermediate, and high risk groups were simulated using data on subsequent treatments following time to recurrence by risk group, [17]–[20] time to mCRPC following disease recurrence (after subsequent treatment), [21] time to PCa death following mCRPC, [22] and time to overall death following active surveillance or disease recurrence/non-recurrence. [28] Patients who progressed to mCRPC were assumed to only die from PCa. [7] For low risk, annual probability of recurrence for all treatments was assumed alike. [18] For intermediate risk, annual probability of recurrence for intensity modulated radiation treatment options was assumed alike. [17] The annual rates and state transition probabilities used to develop the model are summarized in Tables 1 and 2. Life expectancy and Health-Adjusted Life Expectancy (HALE) was predicted by the model. HALE was predicted by weighting survival in a specific health state with the following utilities: short-term morbidities (0.88), long-term morbidities (0.90), metastatic castrate resistant (0.85), and end-of-life (0.50) [29].

**Table 1:** Treatment distribution by risk groups.

Active surveillance/treatments	Annual rate	Refs
* Low risk*		
Active surveillance	0.10	[24], [26]
Delayed treatments following active surveillance	0.08, 1–2 years	[18]
	0.04, 3–5 years	[18]
	0.02, 6–10 years	[18]
Radical prostatectomy	0.30	[18], [26]
Intensity modulated radiation therapy	0.30	[18], [26]
Brachytherapy	0.30	[17], [26]
* Intermediate risk*		
Radical prostatectomy	0.49	[17], [20], [27]
Intensity modulated radiation therapy	0.24	[17], [20]
Intensity modulated radiation therapy+androgen deprivation therapy	0.19	[17], [20]
Intensity modulated radiation therapy+brachytherapy	0.08	[17], [20]
* High risk*		
Intensity modulated radiation therapy+androgen deprivation therapy	0.77	[17], [20]
Intensity modulated radiation therapy+androgen deprivation therapy+brachytherapy	0.23	[17], [20]

**+** multimodal treatment.

**Table 2:** Health state transition probabilities.

Health state	Annualprobability	Refs
Active treatment → PCa recurrence		
* Low risk*		
Active surveillance → PCa recurrence	0.14	[18]
Radical prostatectomy → PCa recurrence	0.03	[18]
Intensity modulate radiation therapy → PCa recurrence	0.03	[18]
Brachytherapy → PCa recurrence	0.03	[18]
* Intermediate risk*		
Radical prostatectomy → PCa recurrence	0.03	[19]
Intensity modulated radiation therapy therapy→ PCa recurrence	0.04	[17]
Intensity modulated radiation therapy+brachytherapy → PCa recurrence	0.04	[17]
Intensity modulated radiation therapy+androgen deprivation therapy→ PCa recurrence	0.04	[19]
* High risk*		
Intensity modulate radiation therapy+androgen deprivation therapy → PCa recurrence	0.09	[19]
Intensity modulate radiation therapy+androgen deprivation therapy+brachytherapy → PCa recurrence	0.08	[20]
On PCa recurrence	1-(P*_mCRPC_*+P*_overall death_*)	[21], [28]
PCa recurrence free → PCa recurrence		
* Low risk*		
Radical prostatectomy → PCa recurrence	0.03	[18]
Intensity modulate radiation therapy → PCa recurrence	0.03	[18]
Brachytherapy → PCa recurrence	0.03	[18]
* Intermediate risk*		
Radical prostatectomy → PCa recurrence	0.03	[19]
Intensity modulated radiation therapy therapy→ PCa recurrence	0.04	[17]
Intensity modulated radiation therapy+brachytherapy → PCa recurrence	0.04	[17]
Intensity modulated radiation therapy+androgen deprivation therapy → PCa recurrence	0.04	[19]
* High risk*		
Intensity modulate radiation therapy+androgen deprivation therapy → PCa recurrence	0.09	[19]
Intensity modulate radiation therapy+androgen deprivation therapy+brachytherapy) → PCa recurrence	0.08	[20]
On PCa recurrence free	1-(P*_recurrence_*+P*_overall death_*)	[17]–[20], [28]
On active surveillance	1-(P*_active tx_* +P*_overall death_*)	[18], [28]
Active surveillance, PCa recurrence, or PCa recurrence free → overall death	0.02, 1–5 years	[28]
	0.03, 6–10 years	[28]
	0.04, 11–15 years	[28]
	0.07, 16–20 years	[28]
	0.12, ≥21 years	[28]
PCa recurrence → metastatic castrate resistant prostate cancer	0.07	[21]
On metastatic castrate resistant prostate cancer	0.73	[22]
Metastatic castrate resistant prostate cancer → PCa death	0.27	[22]

PCa – prostate cancer,+multimodal treatment, P*_mCRPC -_* probability of metastatic castrate resistant prostate cancer, P*_overall death_* - probability of overall death, P*_recurrence_* - probability of cancer recurrence, P*_active tx_* – probability of active treatment.

The disease management trajectories specific to each patient was simulated using the Monte Carlo microsimulation. In the microsimulation, the underlying hypothetical cohort was estimated with each simulated patient proceeding through the model individually. The simulation involved trails with patients making random walks from PCa diagnosis to end-of-life represented in Figure 1. This iteration was repeated over the specific period of time (5-, 10-, 15-year and lifetime period), and once completed the next patient transited through the model. Each patient encountered distinct disease evolution trajectories ascertained by their state transition probabilities during the simulation. [23] Incident events that occurred during the simulation were counted by tracker variables. Tracker variables added memory to the Markov structure. [23] The model was developed in TreeAge Pro Suite (TreeAge Software Inc., Williamstown, MA, USA) [30].

## Analyses

### Validation of the model

Internal validation examined model’s internal consistency and assumptions at the population level [31]. The model predicted rates on treatments for PCa recurrence by risk group at diagnosis, mCRPC, overall and PCa deaths were compared with rates derived from peer-reviewed literature used to develop the model. Predicted annual rates and observed annual rates were compared with t-tests. A two sided p-value of 0.05 was set as the level of significance.

### Sensitivity analyses

Sensitivity analyses were performed to examine robustness of model findings. One-way sensitivity analyses were performed by varying the input value of a parameter at a time while the rest were held at their base case values (Table 3). Following transition probabilities of base case were varied over values reported in the literature: (i) low risk cohort received primary androgen deprivation therapy, (ii) active treatment distribution, (iii) PCa recurrence following initial treatments, (iv) PCa death following mCRPC, and (v) overall death following active surveillance or PCa recurrence free. Two-way analysis assessed clinically relevant interaction between parameters and its bearing on survival.

**Table 3:** Annual rates and probabilities for base case and sensitivity analyses.

Health state	Base case[refs]	Sensitivityanalyses [refs]
Active surveillance/treatments		
* Low risk*		
Active surveillance	0.10 [24], [26]	0.20 [25]
Delayed treatments following active surveillance	0.08, 1–2 years [18]	0.10, 1–2 years [44]
	0.04, 3–5 years [18]	0.05, 3–5 years [44]
	0.02, 6–10 years [18]	0.02, 6–10 years [44]
Radical prostatectomy	0.30 [18], [26]	0.26 [25]
Intensity modulated radiation therapy	0.30 [18], [26]	0.27 [25]
Brachytherapy	0.30 [18], [26]	0.27 [25]
Primary androgen deprivation therapy	Not applicable	0.06 [25]
* Intermediate risk*		
Radical prostatectomy	0.49 [17], [20], [27]	0.46 [45], [65]
Intensity modulated radiation therapy	0.24 [17], [20]	0.23 [45], [65]
Intensity modulated radiation therapy+androgen deprivation therapy	0.19 [17], [20]	0.21 [42]
Intensity modulated radiation therapy+brachytherapy	0.08 [17], [20]	0.10 [45], [65]
* High risk*		
Intensity modulated radiation therapy+androgen deprivation therapy	0.77 [17], [20]	0.47 [65]
Intensity modulated radiation therapy+androgen deprivation therapy+brachytherapy	0.23 [17], [20]	0.52 [65]
PCa recurrence		
* Low risk*		
Radical prostatectomy → PCa recurrence	0.03 [18]	0.01 [44]
Intensity modulate radiation therapy → PCa recurrence	0.03 [18]	0.05 [44]
Brachytherapy → PCa recurrence	0.03 [18]	0.02 [66]
Androgen deprivation therapy → PCa recurrence	Not applicable	0.01 [44]
* Intermediate risk*		
Radical prostatectomy → PCa recurrence	0.03 [19]	0.05 [37]
Intensity modulated radiation therapy+brachytherapy → PCa recurrence	0.04 [17]	0.01 [67]
Intensity modulated radiation therapy → PCa recurrence	0.04 [17]	0.03 [67]
Intensity modulated radiation therapy+androgen deprivation therapy → PCa recurrence	0.04 [19]	0.03 [68]
* High risk*		
Intensity modulate radiation therapy+androgen deprivation therapy → PCa recurrence	0.09 [19]	0.07 [68]
Intensity modulate radiation therapy+androgen deprivation therapy+brachytherapy → PCa recurrence	0.08 [20]	0.10 [69]
Active surveillance, PCa recurrence, or PCa recurrence free → overall death	0.02, 1–5 years [28]	0.02, 1–5 years [70], [71]
	0.03, 6–10 years [28]	0.03, 6–10 years [70], [71]
	0.04, 11–15 years [28]	0.04, 11–15 years [70], [71]
	0.07, 16–20 years [28]	0.07, 16–20 years [70], [71]
	0.12, ≥21 years [28]	0.15, ≥21 years [70], [71]
Metastatic castrate resistant prostate cancer → PCa death	0.27 [22]	0.35 [72]

PCa – prostate cancer,+multimodal treatment, Not applicable – treatment option not considered for base case.

### Outcome assessment

The model predicted clinical outcomes were: rate of recurrence following initial treatment, rate of mCRPC, rate of PCa death and overall death. These rates were predicted for the overall cohort, by risk groups and initial treatment strategies over specified time periods. Monte Carlo microsimulations of 1000 samples were used to stabilize model predicted estimate (e.g. mean) and the variability in results across simulated cohorts generated the 95% confidence interval (95% CI) [32].

## Results

### Model validation

Validation demonstrated good internal consistency of the model. The outcomes predicted by overall, low, intermediate, and high risk cohorts were similar to the observed outcomes derived from the literature, (p = 0.49), (p = 0.62), (p = 0.47), (p = 0.51), respectively. The annual rates predicted by the model were comparable to the observed annual rates derived from the literature (Table 4). The model predicted outcomes demonstrated good concordance with the disease evolution and observed outcomes.

**Table 4:** Model validation.

Health state	Predictedannual rate	Observedannual rate (refs)	p-value
PCa recurrence			
* Low risk*			
Active surveillance → PCa recurrence	0.12	0.15 [18]	0.21
Radical prostatectomy → PCa recurrence	0.02	0.03 [18]	0.24
Intensity modulate radiation therapy → PCa recurrence	0.04	0.03 [18]	0.31
Brachytherapy → PCa recurrence	0.02	0.03 [18]	0.17
* Intermediate risk*			
Radical prostatectomy → PCa recurrence	0.04	0.03 [19]	0.15
Intensity modulated radiation therapy+brachytherapy → PCa recurrence	0.05	0.04 [17]	0.11
Intensity modulated radiation therapy+androgen deprivation therapy → PCa recurrence	0.06	0.04 [19]	0.10
Intensity modulated radiation therapy → PCa recurrence	0.05	0.04 [17]	0.14
* High risk*			
Intensity modulate radiation therapy+androgen deprivation therapy → PCa recurrence	0.11	0.09 [19]	0.12
Intensity modulate radiation therapy+androgen deprivation therapy+brachytherapy → PCa recurrence	0.07	0.08 [20]	0.30
PCa recurrence → metastatic castrate resistant prostate cancer	0.05	0.08 [21]	0.39
Metastatic castrate resistant prostate cancer → PCa death	0.29	0.31 [22]	0.16

PCa – prostate cancer; predicted annual rate – model predicted; observed annual rate – annual rates derived from the literature.

### Sensitivity analyses

One-way sensitivity analyses demonstrated marginal variation in the rate of outcomes across various scenarios considered (Table 5). These results underscore the robustness of base case findings. When both the annual probability of cancer recurrence and mCRPC was varied (i.e. two-way sensitivity analysis) PCa deaths increased compared to base case; 3.2% (95%CI 2.5%–3.9%) vs. 2.4% (95%CI 1.7%–3.1%), and 9.4% (95%CI 8.3%–10.5%) vs. 6.3% (95%CI 5.8%–6.8%) over 10-, and 15-year, respectively.

**Table 5:** Sensitivity analyses.

	% PCa recurrence (95%CI)	% mCRPC (95%CI)	% PCa death (95%CI)	% Overall death (95%CI)
**5-year**				
**Base case**	**13.2 (11.8–14.6)**	**0.7 (0.5–1.0)**	**0.0**	**7.6 (5.4–9.8)**
Sensitivity analysis I	12.1 (11.3–12.9)	0.5 (0.2–0.8)	0.0	7.3 (6.5–8.1)
Sensitivity analysis II	15.9 (15.2–16.6)	0.9 (0.6–1.2)	0.0	8.5 (7.8–9.2)
Sensitivity analysis III	11.2 (10.6–11.8)	0.6 (0.3–0.9)	0.0	8.1 (7.5–8.7)
Sensitivity analysis IV	14.6 (13.9–15.3)	1.1 (0.9–1.3)	0.0	8.3 (7.8–8.8)
Sensitivity analysis V	12.8 (11.7–13.9)	0.8 (0.5–1.1)	0.0	8.2 (7.1–9.4)
**10-year**				
**Base case**	**20.1 (18.5–21.7)**	**5.1 (4.3–5.9)**	**2.4 (1.7–3.1)**	**18.1 (16.5–19.7)**
Sensitivity analysis I	19.6 (19.1–20.1)	5.2 (4.7–5.7)	2.3 (1.5–3.1)	17.4 (16.9–17.9)
Sensitivity analysis II	23.5 (22.8–24.2)	6.9 (6.2–7.6)	3.1 (2.3–3.9)	20.3 (19.4–21.2)
Sensitivity analysis III	17.4 (16.3–18.6)	4.3 (3.5–5.1)	1.5 (0.9–2.1)	19.6 (19.1–20.1)
Sensitivity analysis IV	22.3 (21.7–22.9)	5.8 (5.5–6.2)	2.7 (2.5–2.9)	19.4 (18.7–20.1)
Sensitivity analysis V	19.5 (18.7–20.3)	4.9 (4.5–5.3)	2.2 (1.8–2.6)	20.6 (20.3–20.9)
**15-year**				
**Base case**	**27.8 (27.1–28.5)**	**9.2 (8.3–10.1)**	**6.3 (5.4–7.2)**	**31.4 (29.6–33.2)**
Sensitivity analysis I	27.6 (26.9–28.3)	9.4 (8.9–9.9)	5.8 (4.9–6.7)	30.2 (29.3–31.1)
Sensitivity analysis II	31.9 (31.2–32.6)	11.3 (10.4–12.2)	7.6 (6.8–8.4)	34.8 (34.2–35.4)
Sensitivity analysis III	25.8 (25.4–26.2)	8.8 (8.4–9.2)	5.4 (5.1–5.8)	33.9 (33.4–34.4)
Sensitivity analysis IV	29.4 (28.3–30.6)	10.3 (9.4–11.2)	8.5 (7.8–9.2)	30.6 (29.5–31.7)
Sensitivity analysis V	27.3 (26.4–28.2)	8.7 (8.2–9.2)	5.1 (4.3–5.9)	34.7 (33.8–35.6)

PCa – prostate cancer, mCRPC – metastatic castrate resistant prostate cancer, 95%CI –95% confidence interval, Sensitivity analysis I - primary androgen deprivation therapy received by low-risk cohort; Sensitivity analysis II - varied rates of active surveillance/treatments for base case; Sensitivity analysis III - varied rates of PCa recurrence/non-recurrence for base case; Sensitivity analysis IV - varied rate of PCa death for base case; Sensitivity analysis V - varied rate of overall death for base case.

### Outcome assessment

The predicted mean life expectancy was 20.9 years (95%CI 20.5 years–21.3 years), 22.8 years (95%CI 22.1 years–23.5 years), 19.6 years (95%CI 18.8 years–20.4 years), and 17.3 years (95%CI 16.5 years–18.1 years) for overall cohort, low, intermediate, and high risk, respectively. The predicted mean HALE was 18.2 years (95% CI 17.9 years–18.5 years), 21.7 years (95%CI 21.1 years–22.3 years), 18.1 years (95%CI 17.3 years–18.9 years), and 13.4 years (95% CI 12.6 years–14.2 years), respectively.

Over the lifetime simulated period, PCa death for overall cohort, low, intermediate, and high risk was14.3% (95%CI 13.1%–15.5%), 1.8% (95%CI 1.2%–2.4%), 16.4% (95%CI 15.6%–17.2%), and 39.6% (95%CI 38.3%–40.9%), respectively. Similarly, overall death was 85.7% (95%CI 83.5%–87.9%), 98.2% (95%CI 97.3%–99.1%), 83.6% (95%CI 81.9%–85.3%), and 60.4% (95%CI 57.3%–63.5%), respectively. Tables 6 and 7 summarize the predicted outcomes by risk groups and treatment strategies. Figures S1-S4 illustrates the distribution of clinical outcomes by simulated cohorts.

**Table 6:** Predicted outcomes by risk groups.

	% PCa recurrence (95%CI)	% mCRPC (95%CI)	% PCa death (95%CI)	% Overall death (95%CI)
**5-year**				
Overall cohort	13.2 (11.8–14.6)	0.7 (0.5–0.9)	0.0	7.6 (5.4–9.8)
Low risk	9.7 (9.1–10.3)	0.0	0.0	5.4 (4.7–6.1)
Intermediate risk	12.6 (11.3–13.9)	0.0	0.0	8.5 (7.6–9.4)
High risk	19.4 (17.6–21.2)	3.8 (2.9–4.7)	0.0	10.9 (10.1–11.7)
**10-year**				
Overall cohort	20.1 (18.5–21.7)	5.1 (4.3–5.9)	2.4 (1.7–3.1)	18.1 (16.5–19.7)
Low risk	14.3 (13.7–14.9)	0.0	0.0	13.5 (13.1–13.9)
Intermediate risk	21.5 (20.4–22.6)	2.8 (2.1–3.5)	0.0	18.9 (17.5–20.3)
High risk	30.2 (29.1–31.3)	20.3 (18.7–21.9)	8.7 (8.1–9.3)	24.6 (23.5–25.7)
**15-year**				
Overall cohort	27.8 (27.1–28.5)	9.2 (8.3–10.1)	6.3 (5.4–7.2)	31.4 (29.6–33.2)
Low risk	20.3 (19.7–20.9)	0.0	0.0	23.2 (22.1–24.3)
Intermediate risk	32.1 (31.7–32.5)	6.1 (5.4–6.8)	2.8 (1.9–3.7)	30.1 (29.4–30.8)
High risk	42.6 (41.4–43.8)	33.5 (32.7–34.3)	19.1 (17.9–20.3)	39.2 (37.9–40.5)

PCa – prostate cancer, mCRPC – metastatic castrate resistant prostate cancer, 95%CI –95% confidence interval**.**

**Table 7:** Predicted outcomes by treatment strategies.

	% PCa recurrence (95%CI)	% mCRPC (95%CI)	% PCa death (95%CI)	% Overall death (95%CI)
**Low risk**				
**5-year**				
AS	6.3 (5.9–6.7)	0.0	0.0	4.5 (4.1–4.9)
RP	9.5 (9.2–9.8)	0.0	0.0	5.6 (4.9–6.3)
BT	9.9 (9.6–10.2)	0.0	0.0	5.9 (5.4–6.4)
IMRT	11.2 (10.7–11.7)	0.0	0.0	6.5 (5.7–7.3)
**10-year**				
AS	11.4 (10.8–11.8)	0.0	0.0	8.7 (8.2–9.2)
RP	15.3 (14.9–15.7)	0.0	0.0	13.6 (13.1–14.1)
BT	16.1 (15.4–16.8)	0.0	0.0	14.2 (13.8–14.6)
IMRT	17.5 (16.6–18.4)	0.0	0.0	15.8 (15.1–16.5)
**15-year**				
AS	17.2 (16.6–17.8)	0.0	0.0	15.2 (14.5–15.9)
RP	21.5 (20.9–22.1)	0.0	0.0	24.5 (23.7–25.3)
BT	22.6 (22.3–22.9)	0.0	0.0	25.3 (24.5–26.1)
IMRT	24.5 (23.8–25.2)	0.0	0.0	27.6 (26.8–28.4)
**Intermediate risk**				
**5-year**				
RP	10.6 (9.9–11.3)	0.0	0.0	6.8 (6.4–7.2)
IMRT	12.2 (11.6–12.8)	0.0	0.0	9.2 (8.6–9.8)
IMRT+ADT	14.1 (13.3–14.9)	0.0	0.0	10.7 (10.1–11.3)
IMRT+BT	11.3 (10.5–12.1)	0.0	0.0	7.5 (6.9–8.1)
**10-year**				
RP	17.2 (16.1–18.3)	1.9 (1.6–2.2)	0.0	14.5 (13.1–15.9)
IMRT	19.3 (17.9–20.7)	2.6 (2.1–3.1)	0.0	19.7 (18.1–21.3)
IMRT+ADT	21.6 (19.9–23.3)	3.2 (2.6–3.8)	0.0	22.6 (20.8–24.4)
IMRT+BT	18.1 (16.8–19.4)	2.1 (1.7–2.5)	0.0	15.4 (14.5–16.3)
**15-year**				
RP	28.9 (27.3–30.5)	4.7 (4.2–5.2)	2.1 (1.8–2.4)	26.3 (24.7–27.9)
IMRT	31.8 (29.9–33.7)	5.8 (5.2–6.4)	3.5 (2.9–4.1)	31.7 (29.3–34.1)
IMRT+ADT	34.6 (32.3–36.9)	6.9 (6.7–7.1)	4.8 (4.2–5.4)	34.8 (32.2–37.4)
IMRT+BT	30.1 (28.4–31.8)	5.2 (4.5–5.9)	2.6 (1.9–3.3)	27.5 (25.4–29.6)
**High risk**				
**5-year**				
IMRT+ADT	19.6 (18.3–20.9)	4.2 (3.5–4.9)	0.0	12.4 (10.6–14.2)
IMRT+ADT+BT	17.5 (16.4–18.6)	2.1 (1.8–2.4)	0.0	8.6 (7.4–9.8)
**10-year**				
IMRT+ADT	32.7 (30.9–34.5)	23.5 (21.9–25.1)	11.5 (9.8–13.2)	26.4 (24.2–28.6)
IMRT+ADT+BT	28.6 (27.3–29.9)	16.7 (15.6–17.8)	6.9 (5.7–8.1)	23.5 (21.7–25.3)
**15-year**				
IMRT+ADT	45.2 (43.8–46.6)	36.4 (34.2–38.6)	23.5 (21.2–25.8)	40.5 (37.4–43.6)
IMRT+ADT+BT	37.3 (36.1–38.5)	30.1 (28.5–31.7)	17.4 (15.6–19.2)	38.6 (36.3–40.9)

AS-active surveillance, RP-radical prostatectomy, BT-brachytherapy, IMRT-intensity modulated radiation therapy, ADT-androgen deprivation therapy, mCRPC-metastatic castrate resistant prostate cancer, PCa-prostate cancer, 95%CI –95% confidence interval,+multimodal treatment.

## Discussion

This study delineates for the first time the development, validation, and outcomes predicted by a simulation model for the contemporary management of PCa from diagnosis to the end-of-life. Internal validation demonstrated good internal consistency of the model whereas sensitivity analyses indicated robustness of base case findings. Of note, the findings reported by this study extend to include long-term forecasted outcomes over 15-years whereas no comparable data exist in the literature. It would be of interest to verify if the long-term follow up of cohorts would concur these predicted rates. This model differed from its predecessors on various key aspects.[9]–[11] Preceding decision analytic models lacked contemporary management options such as active surveillance, intensity modulated radiation therapy, and systemic treatments for mCRPC.[5], [8]–[12] Further, preceding models embraced Markov cohort simulation framework that is memory less to simulate a hypothetical cohort at risk of PCa.[9]–[11] In contrast the current model, (i) from a clinical perspective, this study simulated the contemporary management options of PCa and its bearing on the clinical burden of the disease, and (ii) from a methodological perspective, a Markov model with Monte Carlo microsimulation framework was used. Moreover, the microsimulation with tracker variables overcame the memory less property of Markov cohort simulation embraced by preceding models.[9]–[11] Further, tracker variables enabled individual patient level simulation by integrating transition probabilities based on disease evolution [23].

The contemporary life expectancy at 65 years predicted by the model was comparable to life expectancy reported for Canadian men and other developed nations (17.8–19.3 years) for 2011. [33], [34] The predicted HALE for 2013 was higher compared to 13.8 years reported for Canadian men in 2005/2007. [35] The model predicted survival at 5- and 10-year was comparable to contemporary studies.[36]–[43] Study findings corroborated with the evidence that contemporary management options conferred improved survival. [5], [7], [8] The existing literature lacked studies on clinical outcomes and survival associated with intensity modulated radiation therapy strategies over long follow-up periods (e.g. 15-year) and this prevented adequate comparisons. Further, comparing predicted outcomes was confronted with heterogeneity in reported rates in the literature. This heterogeneity potentially stemmed from patient characteristics (e.g. age, clinical, pathological parameters, and preferences), definition of outcomes, clinical practice, and length of follow-up [19], [40], [44]–[49].

This simulation synthesized evidence on contemporary treatment strategies pertaining to low, intermediate, and high risk groups. For *low risk*, active surveillance conferred improved clinical outcomes and overall survival compared to active treatments. Clinical outcomes and survival were comparable between radical prostatectomy and brachytherapy followed by intensity modulated radiation therapy in the low risk group. These differences potentially stemmed from disparity in patient characteristics specific to treatment options in the low risk group.[19], [44]–[46], [50], [51] For *intermediate group*, the outcomes and survival associated with radical prostatectomy were comparable to intensity modulated radiation therapy+brachytherapy followed by intensity modulated radiation therapy used as monotherapy, and intensity modulated radiation therapy+androgen deprivation therapy. Patients selected for radical prostatectomy compared to radiation therapies were relatively younger with a less severe disease that may explain the difference in predicted outcomes and survival. [45], [46], [52] For this group and *high risk* category, addition of brachytherapy to intensity modulated radiation therapy+androgen deprivation therapy improved clinical outcomes and survival compared to intensity modulated radiation therapy+androgen deprivation therapy. These findings are in agreement with preceding studies reporting that addition of brachytherapy to external beam radiation/intensity modulated radiation therapy might have conferred better clinical outcomes and survival. [20], [53] The predicted overall survival associated with intensity modulated radiation therapy+androgen deprivation was marginally decreased compared to other multimodal treatment options with intensity modulated radiation. This disparity potentially stemmed from androgen deprivation that may exacerbate cardiometabolic risks and potentially lead to marginal increase in overall mortality.[54]–[60] The outcomes associated with treatment options predicted by the model should be generalized with caution since data used to develop the model was retrieved from studies showing differences between treatment groups. Moreover, this study was designed to integrate contemporary treatment options to develop a new decision analytic model and not to evaluate the effectiveness.

There were potential limitations associated with the development of the simulation model. First, assumptions were considered to overcome limitations of the existing literature on observed rates and thereby affect predicted rates. Second, methodological limitations to studies used to develop and validate the model potentially influence the accuracy of state-transition probabilities and predicted outcomes. Third, variation in the epidemiology of the disease, adoption (and reimbursement) of health technologies, and clinical practice across geographic regions limit the generalizability of study findings to healthcare systems from which the model input data was not garnered. However, such a limitation is akin to other disease models.[61]–[64] Finally, management complications associated with treatment choices were not accounted by the model.

In conclusion, this study concurrently integrated the evidence from a wide range of contemporary treatment options to manage PCa to generate a new model where predicted rates corroborated observed rates. Study findings demonstrated contemporary PCa management options conferred life expectancy to patients comparable to general population in Canada and other developed nations. This validated model could be used to assess long-term effectiveness of various PCa management strategies. The flexible structure of the model would permit evaluation of outcomes associated with these health technologies in diverse cohorts. This simulation based study identified limitations to the existing clinical literature. Clinical decision making will greatly benefit from simulation based study given the absence of empirical studies that concurrently evaluated active surveillance and contemporary treatment options for low, intermediate, and high risk PCa from diagnosis to end-of-life.

## Supporting Information

Figure S1 Recurrence rate by simulated cohorts over 5-, 10-, and 15-year.
(TIF)

Figure S2 mCRPC rate by simulated cohorts over 5-, 10-, and 15-year. mCRPC- metastatic castrate resistant prostate cancer.
(TIF)

Figure S3 Mortality rate by simulated cohorts over 5-, 10-, and 15-year.
(TIF)

Figure S4 Mortality by simulated cohorts over lifetime.
(TIF)

## References

[1] Jemal A, Bray F. Center MM, Ferlay J, Ward E, et al (2011) Global cancer statistics. CA Cancer J Clin 61:69–90. 21296855 10.3322/caac.20107

[2] Canadian Cancer Statistics (2013) Available at http://www.cancer.ca/en/cancer-information/cancer-101/canadian-cancer-statistics-publication/?region=qc. Accessed on August 14, 2013.

[3] Rodrigues G, Warde P, Pickles T, Crook J, Brundage M, et al (2012) Pre-treatment risk stratification of prostate cancer patients: A critical review. Can Urol Assoc J 6:121–127. 22511420 10.5489/cuaj.11085 PMC3328553

[4] Hotte SJ, Saad F (2010) Current management of castrate-resistant prostate cancer. Curr Oncol 17 Suppl 2S72–79. 20882137 10.3747/co.v17i0.718 PMC2935714

[5] Saad F, Hotte S, Catton C, Drachenberg D, Finelli A, et al (2013) CUA-CUOG guidelines for the management of castration-resistant prostate cancer (CRPC): 2013 update. Can Urol Assoc J 7:231–237. 24032056 10.5489/cuaj.1542 PMC3758937

[6] Oudard S (2012) Progress in emerging therapies for advanced prostate cancer. Cancer Treat Rev 39:275–289. 23107383 10.1016/j.ctrv.2012.09.005

[7] American Urological Association guideline for the management of Castration Resistant Prostate Cancer. Available at http://www.auanet.org/education/guidelines/castration-resistant-prostate-cancer.cfm. Accessed on July 8, 2013.

[8] National Comprehensive Cancer Network Guidelines. Available at http://www.nccn.org/professionals/physician_gls/f_guidelines.asp#site. Accessed on August 14, 2013.

[9] Grover SA, Coupal L, Zowall H, Rajan R, Trachtenberg J, et al (2000) The clinical burden of prostate cancer in Canada: forecasts from the Montreal Prostate Cancer Model. CMAJ 162:977–983. 10763395 PMC1232349

[10] Cowen ME, Chartrand M, Weitzel WF (1994) A Markov model of the natural history of prostate cancer. J Clin Epidemiol 47:3–21. 8283192 10.1016/0895-4356(94)90029-9

[11] Fleming C, Wasson JH, Albertsen PC, Barry MJ, Wennberg JE (1993) A decision analysis of alternative treatment strategies for clinically localized prostate cancer. Prostate Patient Outcomes Research Team. Jama 269:2650–2658. 8487449

[12] AlDuhaiby EZ, Breen S, Bissonnette JP, Sharpe M, Mayhew L, et al (2012) A national survey of the availability of intensity-modulated radiation therapy and stereotactic radiosurgery in Canada. Radiat Oncol 7:18. 22309806 10.1186/1748-717X-7-18 PMC3339388

[13] Drummond MF, Sculpher MJ, Torrance GW, O’Brien BJ, Stoddart GL, et al**.** (2005) Methods for the economic evaluation of health care programme. Third edition: Oxford: Oxford University Press.

[14] Hunink MGM, Glasziou PP, Siegel JE, Weeks JC, Pliskin JS, et al**.** (2001) Decision Making in Health and Medicine: Interpreting Evidence and Values. Cambridge: Cambridge University Press.

[15] Ficarra V, Novara G, Artibani W, Cestari A, Galfano A, et al (2009) Retropubic, laparoscopic, and robot-assisted radical prostatectomy: a systematic review and cumulative analysis of comparative studies. Eur Urol 55:1037–1063. 19185977 10.1016/j.eururo.2009.01.036

[16] Pierorazio PM, Mullins JK, Eifler JB, Voth K, Hyams ES, et al**.** (2013) Contemporaneous comparison of open vs minimally-invasive radical prostatectomy for high-risk prostate cancer. BJU Int. 10.1111/j.1464-410X.2012.11757.x PMC3978171 23356390

[17] Vora SA, Wong WW, Schild SE, Ezzell GA, Andrews PE, et al (2013) Outcome and toxicity for patients treated with intensity modulated radiation therapy for localized prostate cancer. J Urol 190:521–526. 23415964 10.1016/j.juro.2013.02.012

[18] Klotz L, Zhang L, Lam A, Nam R, Mamedov A, et al (2009) Clinical results of long-term follow-up of a large, active surveillance cohort with localized prostate cancer. J Clin Oncol 28:126–131. 19917860 10.1200/JCO.2009.24.2180

[19] Aizer AA, Yu JB, Colberg JW, McKeon AM, Decker RH, et al (2009) Radical prostatectomy vs. intensity-modulated radiation therapy in the management of localized prostate adenocarcinoma. Radiother Oncol 93:185–191. 19800702 10.1016/j.radonc.2009.09.001

[20] Kotecha R, Yamada Y, Pei X, Kollmeier MA, Cox B, et al (2012) Clinical outcomes of high-dose-rate brachytherapy and external beam radiotherapy in the management of clinically localized prostate cancer. Brachytherapy 12:44–49. 22831750 10.1016/j.brachy.2012.05.003

[21] Crook JM, O’Callaghan CJ, Duncan G, Dearnaley DP, Higano CS, et al (2012) Intermittent androgen suppression for rising PSA level after radiotherapy. N Engl J Med 367:895–903. 22931259 10.1056/NEJMoa1201546 PMC3521033

[22] Dragomir A, Dinea D, Vanhuyse M, Cury FL, Aprikian AG (2014) Drug costs in the management of metastatic castration-resistant prostate cancer in Canada. BMC Health Serv Res 14:252. 24927758 10.1186/1472-6963-14-252 PMC4099156

[23] Siebert U, Alagoz O, Bayoumi AM, Jahn B, Owens DK, et al (2012) State-transition modeling: a report of the ISPOR-SMDM Modeling Good Research Practices Task Force–3. Value Health 15:812–820. 22999130 10.1016/j.jval.2012.06.014

[24] Cooperberg MR, Broering JM, Kantoff PW, Carroll PR (2007) Contemporary trends in low risk prostate cancer: risk assessment and treatment. J Urol 178:S14–19. 17644125 10.1016/j.juro.2007.03.135 PMC2987559

[25] Carter HB (2012) Active surveillance for prostate cancer: an underutilized opportunity for reducing harm. J Natl Cancer Inst Monogr 2012:175–183. 23271770 10.1093/jncimonographs/lgs036 PMC3540867

[26] Keegan KA, Dall’Era MA, Durbin-Johnson B, Evans CP (2011) Active surveillance for prostate cancer compared with immediate treatment: an economic analysis. Cancer 118:3512–3518. 22180322 10.1002/cncr.26688 PMC3698480

[27] Zelefsky MJ, Eastham JA, Cronin AM, Fuks Z, Zhang Z, et al (2010) Metastasis after radical prostatectomy or external beam radiotherapy for patients with clinically localized prostate cancer: a comparison of clinical cohorts adjusted for case mix. J Clin Oncol 28:1508–1513. 20159826 10.1200/JCO.2009.22.2265 PMC3731893

[28] Mortality, Summary List of Causes. Available at http://www.statcan.gc.ca/pub/84f0209x/84f0209×2008000-eng.pdf. Accessed on October 7, 2013.

[29] Pataky R, Gulati R, Etzioni R, Black P, Chi KN, et al. (2014) Is prostate cancer screening cost-effective? A microsimulation model of prostate-specific antigen-based screening for British Columbia, Canada. Int J Cancer. 10.1002/ijc.28732 PMC4410808 24443367

[30] TreeAge Software Inc, Williamstown MA, USA. Available at https://www.treeage.com/. Accessed on December 7, 2013.

[31] Eddy DM, Hollingworth W, Caro JJ, Tsevat J, McDonald KM, et al (2012) Model transparency and validation: a report of the ISPOR-SMDM Modeling Good Research Practices Task Force–7. Value Health 15:843–850. 22999134 10.1016/j.jval.2012.04.012

[32] Kopec JA, Fines P, Manuel DG, Buckeridge DL, Flanagan WM, et al (2010) Validation of population-based disease simulation models: a review of concepts and methods. BMC Public Health 10:710. 21087466 10.1186/1471-2458-10-710 PMC3001435

[33] Report on the Demographic Situation in Canada. Mortality: Overview, (2000 and 2011) Available at http://www.statcan.gc.ca/pub/91-209-x/2013001/article/11867-eng.htm. Accessed on May 12,2014.

[34] OECD (2013), Health at a Glance 2013: OECD Indicators, OECD Publishing. Available at http://dx.doi.org/10.1787/health_glance-2013-en. Accessed on May 12,2014.

[35] Statistics Canada. Table 102–0122 - Health-adjusted life expectancy, at birth and at age 65, by sex and income, Canada and provinces, occasional (years) Available at http://www5.statcan.gc.ca/cansim/a05. Accessed on May 12,2014.

[36] Alicikus ZA, Yamada Y, Zhang Z, Pei X, Hunt M, et al (2011) Ten-year outcomes of high-dose, intensity-modulated radiotherapy for localized prostate cancer. Cancer 117:1429–1437. 21425143 10.1002/cncr.25467

[37] Roehl KA, Han M, Ramos CG, Antenor JA, Catalona WJ (2004) Cancer progression and survival rates following anatomical radical retropubic prostatectomy in 3,478 consecutive patients: long-term results. J Urol 172:910–914. 15310996 10.1097/01.ju.0000134888.22332.bb

[38] van den Bergh RC, Roemeling S, Roobol MJ, Aus G, Hugosson J, et al (2008) Outcomes of men with screen-detected prostate cancer eligible for active surveillance who were managed expectantly. Eur Urol 55:1–8. 18805628 10.1016/j.eururo.2008.09.007

[39] Astrom L, Pedersen D, Mercke C, Holmang S, Johansson KA (2005) Long-term outcome of high dose rate brachytherapy in radiotherapy of localised prostate cancer. Radiother Oncol 74:157–161. 15734203 10.1016/j.radonc.2004.10.014

[40] Eggener SE, Scardino PT, Walsh PC, Han M, Partin AW, et al (2011) Predicting 15-year prostate cancer specific mortality after radical prostatectomy. J Urol 185:869–875. 21239008 10.1016/j.juro.2010.10.057 PMC4058776

[41] Merino T, San Francisco IF, Rojas PA, Bettoli P, Zuniga A, et al (2013) Intensity-modulated radiotherapy versus radical prostatectomy in patients with localized prostate cancer: long-term follow-up. BMC Cancer 13:530. 24209381 10.1186/1471-2407-13-530 PMC3833713

[42] Galalae RM, Martinez A, Mate T, Mitchell C, Edmundson G, et al (2004) Long-term outcome by risk factors using conformal high-dose-rate brachytherapy (HDR-BT) boost with or without neoadjuvant androgen suppression for localized prostate cancer. Int J Radiat Oncol Biol Phys 58:1048–1055. 15001244 10.1016/j.ijrobp.2003.08.003

[43] Prada PJ, Mendez L, Fernandez J, Gonzalez H, Jimenez I, et al (2012) Long-term biochemical results after high-dose-rate intensity modulated brachytherapy with external beam radiotherapy for high risk prostate cancer. Radiat Oncol 7:31. 22397528 10.1186/1748-717X-7-31 PMC3310720

[44] Tosoian JJ, Trock BJ, Landis P, Feng Z, Epstein JI, et al (2011) Active surveillance program for prostate cancer: an update of the Johns Hopkins experience. J Clin Oncol 29:2185–2190. 21464416 10.1200/JCO.2010.32.8112

[45] D’Amico AV, Whittington R, Malkowicz SB, Schultz D, Blank K, et al (1998) Biochemical outcome after radical prostatectomy, external beam radiation therapy, or interstitial radiation therapy for clinically localized prostate cancer. JAMA 280:969–974. 9749478 10.1001/jama.280.11.969

[46] D’Amico AV, Moul JW, Carroll PR, Sun L, Lubeck D, et al (2003) Surrogate end point for prostate cancer-specific mortality after radical prostatectomy or radiation therapy. J Natl Cancer Inst 95:1376–1383. 13130113 10.1093/jnci/djg043

[47] Zelefsky MJ, Pei X, Chou JF, Schechter M, Kollmeier M, et al (2011) Dose escalation for prostate cancer radiotherapy: predictors of long-term biochemical tumor control and distant metastases-free survival outcomes. Eur Urol 60:1133–1139. 21889832 10.1016/j.eururo.2011.08.029 PMC4037155

[48] Stephenson AJ, Kattan MW, Eastham JA, Dotan ZA, Bianco FJ Jr, et al (2006) Defining biochemical recurrence of prostate cancer after radical prostatectomy: a proposal for a standardized definition. J Clin Oncol 24:3973–3978. 16921049 10.1200/JCO.2005.04.0756

[49] Han M, Partin AW, Pound CR, Epstein JI, Walsh PC (2001) Long-term biochemical disease-free and cancer-specific survival following anatomic radical retropubic prostatectomy. The 15-year Johns Hopkins experience. Urol Clin North Am 28:555–565. 11590814 10.1016/s0094-0143(05)70163-4

[50] D’Amico AV, Cote K, Loffredo M, Renshaw AA, Schultz D (2002) Determinants of prostate cancer-specific survival after radiation therapy for patients with clinically localized prostate cancer. J Clin Oncol 20:4567–4573. 12454114 10.1200/JCO.2002.03.061

[51] Freedland SJ, Humphreys EB, Mangold LA, Eisenberger M, Dorey FJ, et al (2005) Risk of prostate cancer-specific mortality following biochemical recurrence after radical prostatectomy. JAMA 294:433–439. 16046649 10.1001/jama.294.4.433

[52] Sooriakumaran P, Nyberg T, Akre O, Haendler L, Heus I, et al (2014) Comparative effectiveness of radical prostatectomy and radiotherapy in prostate cancer: observational study of mortality outcomes. BMJ 348:g1502. 24574496 10.1136/bmj.g1502 PMC3936107

[53] Sathya JR, Davis IR, Julian JA, Guo Q, Daya D, et al (2005) Randomized trial comparing iridium implant plus external-beam radiation therapy with external-beam radiation therapy alone in node-negative locally advanced cancer of the prostate. J Clin Oncol 23:1192–1199. 15718316 10.1200/JCO.2005.06.154

[54] Efstathiou JA, Bae K, Shipley WU, Hanks GE, Pilepich MV, et al (2008) Cardiovascular mortality and duration of androgen deprivation for locally advanced prostate cancer: analysis of RTOG 92-02. Eur Urol 54:816–823. 18243498 10.1016/j.eururo.2008.01.021

[55] Smith MR, Bae K, Efstathiou JA, Hanks GE, Pilepich MV, et al (2008) Diabetes and mortality in men with locally advanced prostate cancer: RTOG 92-02. J Clin Oncol 26:4333–4339. 18779620 10.1200/JCO.2008.16.5845 PMC2653118

[56] Keating NL, O’Malley AJ, Freedland SJ, Smith MR (2009) Diabetes and cardiovascular disease during androgen deprivation therapy: observational study of veterans with prostate cancer. J Natl Cancer Inst 102:39–46. 19996060 10.1093/jnci/djp404 PMC3107568

[57] Keating NL, O’Malley AJ, Smith MR (2006) Diabetes and cardiovascular disease during androgen deprivation therapy for prostate cancer. J Clin Oncol 24:4448–4456. 16983113 10.1200/JCO.2006.06.2497

[58] Azoulay L, Yin H, Benayoun S, Renoux C, Boivin JF, et al (2011) Androgen-deprivation therapy and the risk of stroke in patients with prostate cancer. Eur Urol 60:1244–1250. 21908097 10.1016/j.eururo.2011.08.041

[59] Bensimon L, Yin H, Suissa S, Pollak MN, Azoulay L (2014) Type 2 diabetes and the risk of mortality among patients with prostate cancer. Cancer Causes Control 25:329–338. 24384808 10.1007/s10552-013-0334-6

[60] Yu O, Eberg M, Benayoun S, Aprikian A, Batist G, et al (2013) Use of statins and the risk of death in patients with prostate cancer. J Clin Oncol 32:5–11. 24190110 10.1200/JCO.2013.49.4757

[61] Drummond MF, Bloom BS, Carrin G, Hillman AL, Hutchings HC, et al (1992) Issues in the cross-national assessment of health technology. Int J Technol Assess Health Care 8:671–682. 1464487 10.1017/s0266462300002361

[62] Hogendoorn W, Schlosser FJ, Moll FL, Muhs BE, Hunink MG, et al (2013) Decision analysis model of open repair versus endovascular treatment in patients with asymptomatic popliteal artery aneurysms. J Vasc Surg 59:651–662. 24246533 10.1016/j.jvs.2013.09.026

[63] Siebert U, Sroczynski G, Hillemanns P, Engel J, Stabenow R, et al (2006) The German cervical cancer screening model: development and validation of a decision-analytic model for cervical cancer screening in Germany. Eur J Public Health 16:185–192. 16469759 10.1093/eurpub/cki163

[64] van Kempen BJ, Ferket BS, Hofman A, Steyerberg EW, Colkesen EB, et al (2012) Validation of a model to investigate the effects of modifying cardiovascular disease (CVD) risk factors on the burden of CVD: the rotterdam ischemic heart disease and stroke computer simulation (RISC) model. BMC Med 10:158. 23217019 10.1186/1741-7015-10-158 PMC3566939

[65] Spratt DE, Pei X, Yamada J, Kollmeier MA, Cox B, et al (2013) Long-term survival and toxicity in patients treated with high-dose intensity modulated radiation therapy for localized prostate cancer. Int J Radiat Oncol Biol Phys 85:686–692. 22795805 10.1016/j.ijrobp.2012.05.023 PMC5705018

[66] Zelefsky MJ, Kuban DA, Levy LB, Potters L, Beyer DC, et al (2006) Multi-institutional analysis of long-term outcome for stages T1-T2 prostate cancer treated with permanent seed implantation. Int J Radiat Oncol Biol Phys 67:327–333. 17084558 10.1016/j.ijrobp.2006.08.056

[67] Spratt DE, Zumsteg ZS, Ghadjar P, Kollmeier MA, Pei X, et al. (2014) Comparison of high-dose (86.4 Gy) IMRT vs combined brachytherapy plus IMRT for intermediate-risk prostate cancer. BJU Int. 10.1111/bju.12514 24447404

[68] Deutsch I, Zelefsky MJ, Zhang Z, Mo Q, Zaider M, et al (2010) Comparison of PSA relapse-free survival in patients treated with ultra-high-dose IMRT versus combination HDR brachytherapy and IMRT. Brachytherapy 9:313–318. 20685176 10.1016/j.brachy.2010.02.196

[69] Stone NN, Stock RG, Cesaretti JA, Unger P (2009) Local control following permanent prostate brachytherapy: effect of high biologically effective dose on biopsy results and oncologic outcomes. Int J Radiat Oncol Biol Phys 76:355–360. 19632069 10.1016/j.ijrobp.2009.01.078

[70] U.S. Population by Age: July 1, 2010. (2010) National Tables and Trends. Available at http://www.aoa.gov/AoARoot/Aging_Statistics/Census_Population/census2010/Index.aspx. Accessed on May 12,2014.

[71] Deaths, Percent of Total Deaths, and Death Rates for the 15 Leading Causes of Death in 5-year Age Groups, by Race and Sex: United States (1999–2010) Available at http://www.cdc.gov/nchs/nvss/mortality/lcwk1.htm. Accessed on May 12,2014.

[72] Tannock IF, de Wit R, Berry WR, Horti J, Pluzanska A, et al (2004) Docetaxel plus prednisone or mitoxantrone plus prednisone for advanced prostate cancer. N Engl J Med 351:1502–1512. 15470213 10.1056/NEJMoa040720

