# Supplementary figures and images for: Clinical Management and Burden of Prostate Cancer: A Markov Monte Carlo Model
Source: PLoS One. 2014 Dec 4;9(12):e113432. doi: 10.1371/journal.pone.0113432 (PMC4256380; doi:10.1371/journal.pone.0113432)

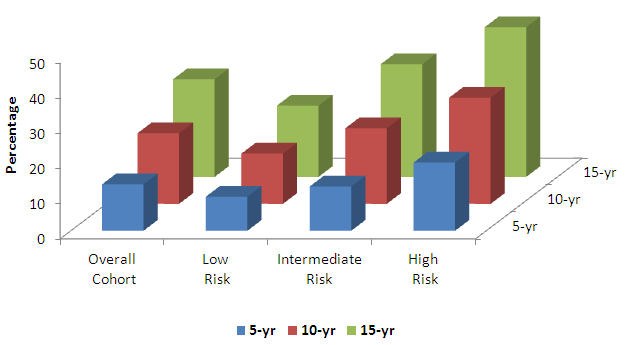

Supplement: Figure S1 — Recurrence rate by simulated cohorts over 5-, 10-, and 15-year. (TIF) [file pone.0113432.s001.tif]

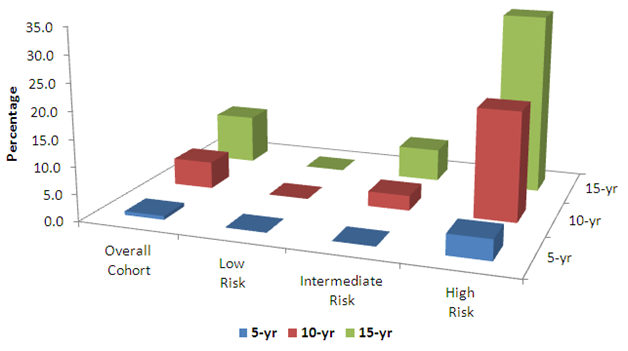

Supplement: Figure S2 — mCRPC rate by simulated cohorts over 5-, 10-, and 15-year. mCRPC- metastatic castrate resistant prostate cancer. (TIF) [file pone.0113432.s002.tif]

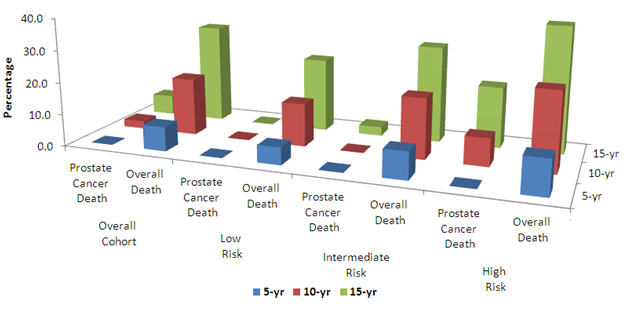

Supplement: Figure S3 — Mortality rate by simulated cohorts over 5-, 10-, and 15-year. (TIF) [file pone.0113432.s003.tif]

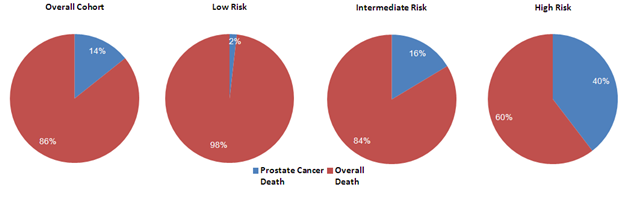

Supplement: Figure S4 — Mortality by simulated cohorts over lifetime. (TIF) [file pone.0113432.s004.tif]
